# Case Report: Walking Pneumonia in Novel Coronavirus Disease (COVID-19): Mild Symptoms with Marked Abnormalities on Chest Imaging

**DOI:** 10.4269/ajtmh.20-0203

**Published:** 2020-04-01

**Authors:** Chaisith Sivakorn, Viravarn Luvira, Sant Muangnoicharoen, Pittaya Piroonamornpun, Tharawit Ouppapong, Anek Mungaomklang, Sopon Iamsirithaworn

**Affiliations:** 1 Department of Clinical Tropical Medicine, Faculty of Tropical Medicine, Mahidol University, Bangkok, Thailand;; 2 Hospital for Tropical Diseases, Faculty of Tropical Medicine, Mahidol University, Bangkok, Thailand;; 3 Division of Epidemiology, Department of Disease Control, Ministry of Public Health, Nonthaburi, Thailand;; 4 Department of Disease Control, Institution for Urban Disease Control and Prevention, Ministry of Public Health, Nonthaburi, Thailand;; 5 Division of Communicable Diseases, Department of Disease Control, Ministry of Public Health, Nonthaburi, Thailand

## Abstract

This case report underlines the appearance of a “walking pneumonia” in a novel coronavirus disease (COVID-19) patient, with evidence of progressive lung involvement on chest imaging studies. The patient traveled from Wuhan, Hubei, China, to Thailand in January 2020. One of her family members was diagnosed with COVID-19. She presented to the hospital because of her concern, but she was without fever or any respiratory symptoms. Three days earlier, her nasopharyngeal and throat swabs revealed a negative severe acute respiratory syndrome coronavirus 2 (SARS-CoV-2) test by real-time reverse transcriptase polymerase chain reaction (RT-PCR). Her initial chest radiography was abnormal, and her first sputum SARS-CoV-2 test yielded inconclusive results. A subsequent sputum test was positive for SARS-CoV-2. Diagnosis in this patient was facilitated by chest imaging and repeat viral testing. Thus, chest imaging studies might enhance capabilities for early diagnosis of COVID-19 pneumonia.

## INTRODUCTION

Since late December 2019, there has been an outbreak of a novel enveloped RNA betacoronavirus^1^ called severe acute respiratory syndrome coronavirus 2 (SARS-CoV-2). This virus causes coronavirus disease 2019 (COVID-19), which has become an ongoing pandemic. The novel coronavirus SARS-CoV-2 is the seventh member of the Coronaviridae family known to infect humans.^1^ The estimated mortality rate of COVID-19 so far is lower than that of severe acute respiratory syndrome or Middle East respiratory syndrome.^2^ However, the ongoing COVID-19 pandemic is a significant health threat worldwide.^3^ We report an important case in which COVID-19 was identified earlier by pneumonia on chest imaging than by clinical symptoms and reverse transcriptase polymerase chain reaction (RT-PCR). Adding this clinical picture of “walking pneumonia” to surveillance case definitions may limit transmission and contribute toward containment of the disease. Furthermore, enhancing the capability of the COVID-19 diagnosis with the use of the chest imaging modality is discussed.

## CASE REPORT

**Figure 1.**
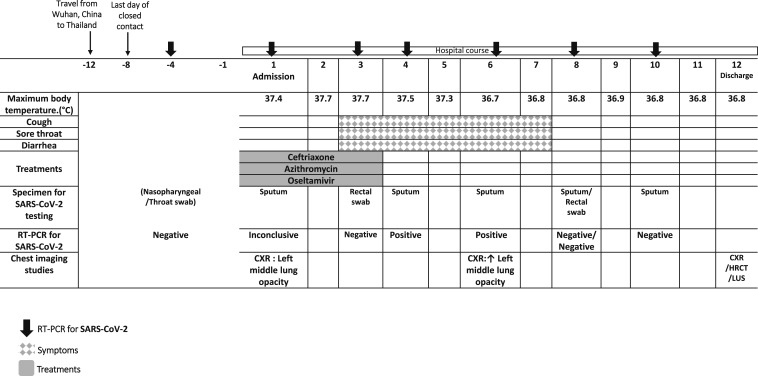
Timeline of exposure and disease course, from January 22, 2020 to February 14, 2020.

A 56-year-old Chinese woman traveled with her family from Wuhan, Hubei, China, to Thailand for leisure on January 22, 2020. Four days later and 8 days before her admission, her husband was admitted to a private hospital after being diagnosed with COVID-19. Four days before her admission, all the other family members including our patient, her two daughters, and her three-year-old grandchild were screened for SARS-CoV-2 from nasopharyngeal and throat swabs using real-time RT-PCR and had negative results (Figure 1). On the day of her admission, she sought health care at our outpatient department because she worried about her condition. She denied history of fever and respiratory symptoms. Physical examination revealed a temperature of 37°C, a pulse rate of 88 beats/minute, a respiratory rate of 20 breaths/minute, a blood pressure of 105/64 mmHg, and an oxygen saturation of 98% while breathing room air. She had no cyanosis, no clubbing, no pursed lips expiration, no use of accessory respiratory muscles, and no nasal flaring. Auscultation of the thorax was normal.

**Figure 2.**
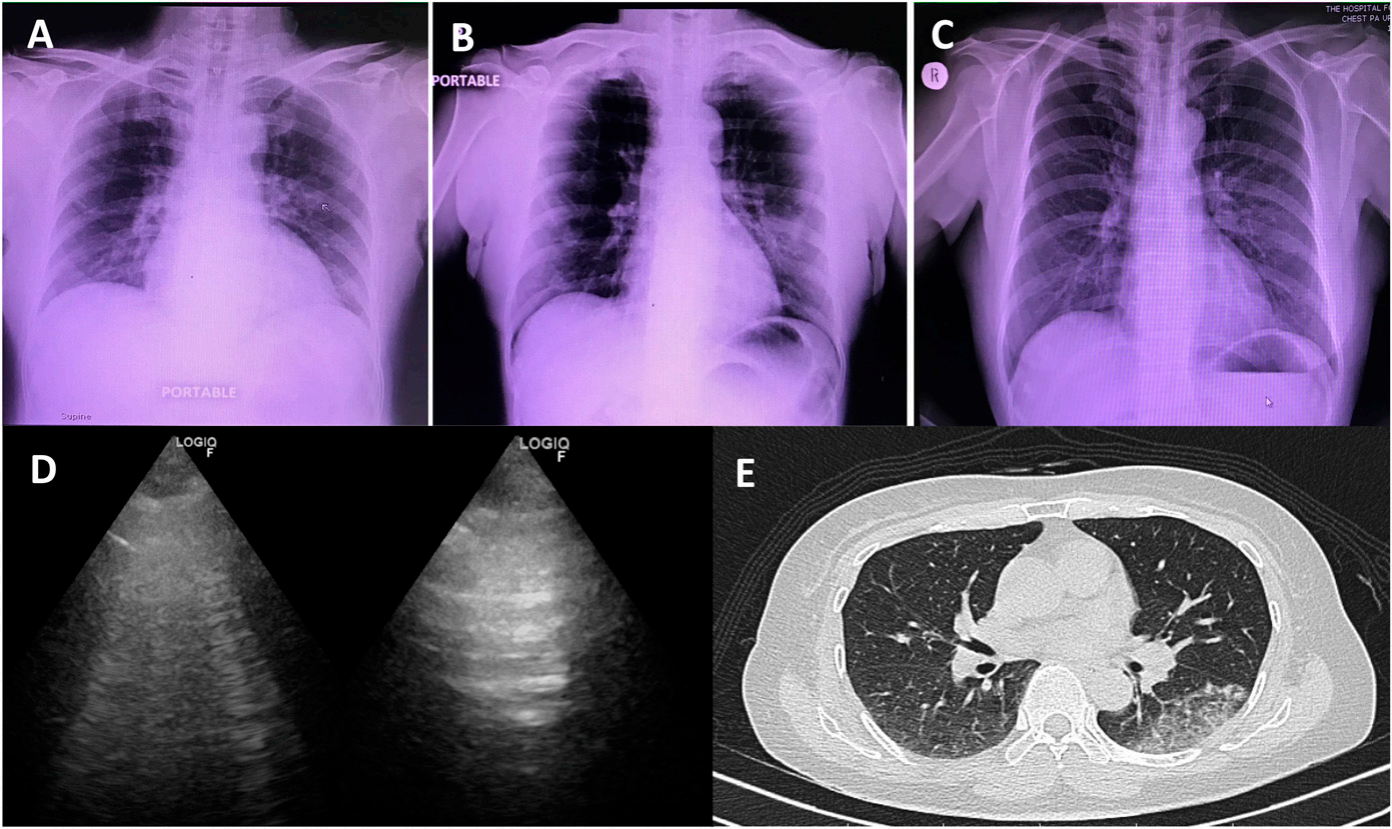
Three modalities of chest imaging studies in coronavirus disease 2019 patient. Chest radiographies (**A**, **B**, and **C**) were obtained on February 3, 8, and 14, 2020; chest ultrasonography and axial high-resolution computed tomography were obtained for the follow-up lung lesion on February 14, 2020.

The history of close contact with one COVID-19 case and the noticeable cough during the physical examination warranted further investigation. Her chest radiography (CXR) revealed an alveolar opacity in the left middle lung field (Figure 2A). Thus, a diagnosis of pneumonia probably due to COVID-19 was made. She was admitted to an airborne infection isolation room, and empirical treatments were started with ceftriaxone, azithromycin, and oseltamivir. Initial blood tests apart from mild leukopenia showed no other abnormalities. Reverse transcriptase polymerase chain reaction of sputum obtained on day 1 of admission was inconclusive for SARS-CoV-2, but sputum obtained on day 4 of admission was positive.^4^ Furthermore, she started to develop sore throat, mild cough, and diarrhea on day 3 of admission (Figure 1). Antibiotic and antiviral treatments were discontinued because RT-PCR for other respiratory viruses and bacteria from sputum was negative. Two rectal swabs were negative for SARS-CoV-2. She continued to receive supportive care and isolation until two consecutive sputum specimens were negative for SARS-CoV-2. All other family members who were previously screened negative remained asymptomatic, but one daughter tested positive for SARS-CoV-2. She was admitted for treatment and isolation in another hospital.

A follow-up CXR obtained on day 6 of admission (Figure 2B) showed progression of the opacity in the left middle lung field and of her symptoms of cough, sore throat, and diarrhea. Further imaging studies were performed for educational purposes on day 12 after admission, when her symptoms were resolved, and the sputum RT-PCR was negative on two consecutive specimens. Chest radiography (Figure 2C) showed improvement of the alveolar opacity in the left middle lung field. Lung ultrasonography (LUS) (Figure 2D) was found to be positive for B lines and dynamic air bronchogram sign at the posterior part of the left middle lung field. A high-resolution computed tomography (HRCT) scan on the same day (Figure 2E) showed a localized subpleural region of ground-glass opacity with superimposed inter- and intralobular septal thickening (crazy paving pattern) at the supero-posterior segment of the left lower lung lobe.

## DISCUSSION

During the initial phase of the COVID-19 outbreak, Thailand implemented temperature and other symptom-based screening of travelers at all points of entry (airport, ports, and ground crossing) along with hospital-based surveillance for symptomatic patients since early January 2020. However, detection of the disease is complicated by the diversity of symptoms and the severity of disease at the time of presentation. This family cluster of cases reflects the real-life situation of screening contact persons and the challenge facing surveillance systems. As we expected, the snapshot single screening of contacts of confirmed cases might be inadequate for those with prolonged exposure, such as in family/household situations. Continuous symptom-based surveillance, self-isolation, and other preventative measures have been implemented to all contact persons for 14 days, leading to early detection of the subsequent cases. The incubation period of the presented case was 8 days, which is much longer than the median incubation period of 4 days reported in the literature.^5^ However, it was still within the 14 days observation period for contact persons.^3^ To date, the disease was confined only to this family, and there have been no new transmissions related to this family cluster.

The presented case report shows the clinical picture of “walking pneumonia” in a COVID-19 patient whose clinical symptoms did not correlate with the evidence of progressive lung involvement demonstrated by multiple chest imaging modalities. This case echoes the latest reports, including the outbreak in a family cluster,^6^ which includes the absence of fever at presentation, the majority of cases demonstrating mild symptoms,^5^ and the utility of chest imaging to facilitate early identification of the disease even in asymptomatic high-risk contacts.^7^ There is also strong evidence that COVID-19 can be transmitted by people who are only mildly ill or even presymptomatic.^8^ Therefore, apart from symptoms and RT-PCR, chest imaging could enhance capabilities for detection of COVID-19 pneumonia among patients with COVID-19 with mild symptoms similar to the presented case.

In China, computed tomography (CT) has been an important imaging modality for assisting the diagnosis and management of patients with COVID-19 pneumonia.^7,9^ Fang et al.^9^ compared the detection rate of initial chest CT and RT-PCR in 51 eventually confirmed COVID-19 cases and reported a higher detection rate for initial CT (98%) than first RT-PCR (71%) (*P* < 0.001).^10^ On admission, the predominant CT findings included ground-glass opacification (GGO), consolidation, bilateral involvement, and peripheral and diffuse distributions.^5^ The CT in the present case was performed after symptom resolution, and our findings were compatible with the late peak to early absorption stage described in a case series of COVID-19 CT findings in 21 confirmed adult Chinese patients.^11^ The overuse of CT may cause some drawbacks including higher radiation exposure and the need for transportation, which increases the risk of disease spreading. Furthermore, the utility of CT as the standard chest imaging study might be inapplicable for resource-limited settings. Because the predominant CT pattern observed in COVID-19 pneumonia on admission is GGO, a CXR is not sensitive to detect this and may demonstrate normal findings in the early stage of infection.^12^ These limitations of both CT and CXR lead to the possibility of using LUS at the bedside as a screening and monitoring tool. It is noninvasive and can be performed at the bedside for those in isolation or in intensive care, thus limiting the risk of spreading the disease compared with transferring patients to CT. Lung ultrasonography is also more readily available in low- to middle-income countries but does need to be performed by trained medical personnel with special precaution. Further research is needed to address the utility of LUS in a diagnostic pathway for patient selection for CT and to explore the application of artificial intelligence in screening chest radiographs in suspected cases.

In conclusion, we report a symptomatically mild COVID-19 case presenting as “walking pneumonia” in which the early diagnosis and management was achieved in the presymptomatic stage by the use of chest imaging studies.
